# Exploring virus-host-environment interactions in a chemotrophic-based underground estuary

**DOI:** 10.1186/s40793-024-00549-6

**Published:** 2024-01-30

**Authors:** Timothy M. Ghaly, Amaranta Focardi, Liam D. H. Elbourne, Brodie Sutcliffe, William F. Humphreys, Paul R. Jaschke, Sasha G. Tetu, Ian T. Paulsen

**Affiliations:** 1 School of Natural Sciences, Macquarie University, Sydney, Australia; 2 Climate Change Cluster (C3), University of Technology Sydney, Sydney, Australia; 3 ARC Centre of Excellence in Synthetic Biology, Macquarie University, Sydney, Australia; 4 NSW Department of Primary Industries, Sydney, Australia; 5 School of Biological Sciences, University of Western Australia, Perth, Australia

**Keywords:** Phages, Viral auxiliary metabolic genes, Biogeochemistry, Phage-host interactions, Subterranean estuary, Virome, Anchialine, Marine oxygen minimum zones

## Abstract

**Background:**

Viruses play important roles in modulating microbial communities and influencing global biogeochemistry. There is now growing interest in characterising their ecological roles across diverse biomes. However, little is known about viral ecology in low-nutrient, chemotrophic-based environments. In such ecosystems, virus-driven manipulation of nutrient cycles might have profound impacts across trophic levels. In particular, anchialine environments, which are low-energy underground estuaries sustained by chemotrophic processes, represent ideal model systems to study novel virus-host-environment interactions.

**Results:**

Here, we employ metagenomic sequencing to investigate the viral community in Bundera Sinkhole, an anchialine ecosystem rich in endemic species supported by microbial chemosynthesis. We find that the viruses are highly novel, with less than 2% representing described viruses, and are hugely abundant, making up as much as 12% of microbial intracellular DNA. These highly abundant viruses largely infect important prokaryotic taxa that drive key metabolic processes in the sinkhole. Further, the abundance of viral auxiliary metabolic genes (AMGs) involved in nucleotide and protein synthesis was strongly correlated with declines in environmental phosphate and sulphate concentrations. These AMGs encoded key enzymes needed to produce sulphur-containing amino acids, and phosphorus metabolic enzymes involved in purine and pyrimidine nucleotide synthesis. We hypothesise that this correlation is either due to selection of these AMGs under low phosphate and sulphate concentrations, highlighting the dynamic interactions between viruses, their hosts, and the environment; or, that these AMGs are driving increased viral nucleotide and protein synthesis via manipulation of host phosphorus and sulphur metabolism, consequently driving nutrient depletion in the surrounding water.

**Conclusion:**

This study represents the first metagenomic investigation of viruses in anchialine ecosystems, and provides new hypotheses and insights into virus-host-environment interactions in such ‘dark’, low-energy environments. This is particularly important since anchialine ecosystems are characterised by diverse endemic species, both in their microbial and faunal assemblages, which are primarily supported by microbial chemosynthesis. Thus, virus-host-environment interactions could have profound effects cascading through all trophic levels.

**Supplementary Information:**

The online version contains supplementary material available at 10.1186/s40793-024-00549-6.

## Introduction

Viruses are considered to be the most abundant and diverse biological entities on Earth, infecting all domains of life, and performing pivotal ecological roles [1, 2]. There is now increasing evidence that viruses are important players in global biogeochemistry [3–6]. This can be through influencing the relative abundance of important biogeochemical-cycling host bacteria and archaea; biophysically redirecting the allocation of nutrient elements during bursts of infection; or through altering host metabolism via the expression of viral auxiliary metabolic genes (AMGs) [3, 6–8].

Phages can indirectly influence nutrient cycles via encoded AMG products. AMGs are highly prevalent among viruses and are involved in diverse functions, including nutrient and lipid metabolism, signalling, transport, cell motility, and biofilm formation [9]. These AMGs likely promote phage production or transmission by reprogramming host physiology and metabolism, which can drastically differ between infected (virocells) versus uninfected cells [10]. There is now a growing interest in surveying the AMGs of viruses from diverse habitats, including marine, freshwater and soil biomes. These investigations have revealed AMGs involved in key biogeochemical processes, including sulphur and thiosulphate oxidation [11], nitrification and denitrification [12], phosphorus metabolism [13], methane oxidation [14] and carbon utilisation [15, 16]. Together, these studies highlight the potentially important role of virus-host-environment interactions in global biogeochemistry.

However, little is known about the ecology of viruses in low-energy, chemotrophic-based environments. Low-nutrient ecosystems are likely to be more susceptible to changes in biogeochemical cycles, which could consequently influence nutrient availability at higher trophic levels. In particular, anchialine environments represent ideal model systems to investigate the role of viruses in oligotrophic ecosystems supported by microbial chemosynthesis. Anchialine systems are defined as tidally-influenced subterranean estuaries that extend inland to the limit of seawater penetration underneath karst or volcanic coastlines [17]. They are generally characterised as dark, low-energy environments, with stratified physicochemical profiles, and are rich in endemic macrofauna and highly novel microbes [17–20]. A significant proportion of metabolic energy that support anchialine fauna is obtained via trophic transfer from microbial chemotrophic processes [21–25]. Anchialine environments have been flagged as ecosystems that may be particularly vulnerable to environmental changes [22], which is especially concerning given that they are rich in endemic species. To date, however, little is known about the role of viruses in anchialine ecosystems, which rely heavily on microbial nutrient cycling.

To investigate the diversity and metabolic potential of an anchialine viral community, we employed shotgun metagenomic sequencing along a depth profile in Bundera Sinkhole, the only known continental anchialine system in the Southern Hemisphere. This sinkhole, which is the only ground opening to the subterranean estuary, is located 1.7 km inland from the Indian Ocean on the karstic coastline of north-western Australia. The sinkhole exhibits a complex physicochemical profile, with increasing salinity and decreasing dissolved oxygen with depth, and polymodal peaks of phosphorus, sulphur and nitrogen compounds [20, 26–28]. It is inhabited by diverse biogeochemical-cycling prokaryotes [20, 25], and endemic invertebrate assemblages, including copepods, remipeds, and polychaetes [29–32]. In the present study, we describe the highly novel and abundant viruses inhabiting Bundera Sinkhole, identify important prokaryote host taxa, and characterise the biogeochemical cycling potential encoded by their AMGs. Notably, several AMGs, involved in promoting nucleotide and protein synthesis, were strongly correlated with a reduction in phosphate and sulphate concentrations in the water. We propose a model for phage-host interactions that drive phosphorus and sulphur metabolism, influencing nutrient cycling and availability in this ecosystem.

## Methods

### Sample collection and metagenomic sequencing

Water physicochemical and metagenomic sequence data were obtained from our previous studies, [20] and [25], respectively. In brief, water sample collection, described in Elbourne, et al. [20], involved pumping water samples from Bundera Sinkhole from depths of 2, 8, 17, 18, 22, and 28 m between the 29th of June and the 1st of July 2015. Water physicochemistry data, including salinity and concentrations of dissolved oxygen (DO), dissolved organic carbon (DOC), iron (Fe), ammonia (NH_3_), nitrate (NO_3_^−^), phosphate (PO_4_^3−^) and sulphate (SO_4_^2−^), were obtained from Elbourne, et al. [20] and are presented in Supplementary Table S1. For metagenomic analysis, ~ 4 L of sampled water were first pre-filtered through 60 μm filters (Millipore Type NY60), before collecting microbial cells on 0.22 μm Sterivex™ filters. This filter size would be expected to remove the majority of unattached phage particles, as the largest known “jumbophage” capsid size is only 0.18 μm [33]. DNA was extracted from the filter-captured microbial cells using the PowerWater® DNA Isolation kit (MO BIO Laboratories, Inc., Carlsbad, USA), according to the manufacturer’s protocol. Metagenomic libraries were prepared for two biological replicates from each depth using the Illumina TruSeq DNA Library Preparation Kit, according to the manufacturer’s protocol, and sequenced on the Illumina HiSeq 2000 platform. Sequencing depth statistics are available as Supplementary Table S2.

The assembled metagenomic sequence data, including binned metagenome-assembled genomes (MAGs), were obtained from Ghaly, et al. [25]. Briefly, this involved co-assembling metagenomes using MEGAHIT v1.2.9 [34, 35], followed by the removal of any contig less than 5,000 bp in length. MAGs were generated using MetaBAT v2.2.15 [36], and quality-filtered using CheckM v1.2.1 [37] and GUNC v1.0.5 [38]. Only MAGs that passed the GUNC chimerism check, and had a CheckM completion > 50% and contamination < 10%, were retained for further analysis. All MIMAG data (minimum information about a metagenome-assembled genome [39]) are available as Supplementary Table S3.

### Viral sequence identification and quality filtering

Following methods proposed by Luo, et al. [9], sequences were considered viral if they were identified by VIBRANT v1.2.0 [40], in standard mode; predicted as high-confidence viral sequences using VirSorter2 v2.2.4 [41] (max_score > = 0.9); and encoded at least one viral marker gene, identified by CheckV (database v1.3) [42]. To reduce false-positive predictions associated with smaller contigs, only contigs greater than 5,000 bp were considered. Putative viral contigs that met these criteria were then clustered at 95% identity into vOTUs with CD-HIT v4.6.8 [43, 44] [parameters: -c 0.95 -s 0.85]. The vOTUs were quality-checked using CheckV, which also removed host regions from prophage contigs. vOTUs were taxonomically classified using PhaGCN v2.0 [45, 46] [parameters: --len 5000].

vOTUs were predicted to be temperate viruses if they were identified as proviruses by CheckV, or harboured lysogeny-specific genes (i.e., integrase, recombinase, excisionase, CI/Cro repressor, or *parAB*) [9]. To detect the lysogeny-specific genes, all vOTU protein sequences were functionally annotated using eggNOG-mapper v2 [47], and manually screened for the above gene annotations. All other vOTUs were considered to be potentially lytic viruses.

### Viral abundance and novelty

Normalised relative abundance of the co-assembled contigs were calculated for each sample using the transcripts per million (TPM) method with CoverM v0.6.1 (https://github.com/wwood/CoverM) in contig mode [parameters: contig -t 24 --coupled -m TPM]. TPM normalisation accounts for both sequencing depth of a sample, and the length of a given assembled sequence, to facilitate relative abundance comparisons across samples [48]. The percentage of each metagenomic sample that was made up of viral DNA was calculated from the TPM sum of all viral contigs divided by 1 million (representing the TPM sum of all contigs).

To estimate the degree of novelty among the viral community, vOTUs were searched, using blastn v2.6.0, against the IMG/VR v3 database [49], containing 18,373 cultivated and 2,314,329 uncultivated viral genomes from diverse biomes. vOTUs were classified as ‘known viruses’ if they displayed > 90% nucleotide identity over at least 70% of either query or database sequence; or ‘similar virus’ if they displayed 50%– 90% nucleotide identity over at least 70% of either query or database sequence. vOTUs that did not meet these criteria were considered to be ‘novel viruses’.

### Viral host range prediction and identification of anti-viral defence systems

The prokaryote hosts of the vOTUs were predicted using the consensus of three approaches: (1) searching for viral sequence fragments contained within host CRISPR spacers; (2) identifying viral-encoded tRNA genes with identical sequences to bacterial/archaeal tRNAs; and (3) using the genomic context of prophages. All contigs were screened for CRISPR arrays using CRISPRidentify v1.1.0 [50] [parameters: --fasta_report True]. All identified CRISPR arrays with a score > 0.4 were searched against vOTUs using blastn v2.13.0 with a reduced word size and without low-complexity filtering [parameters: -word_size 7 -dust no]. Only hits that were between 20 and 75 bp in length, and had less than or equal to one mismatch or gap were retained. Viral tRNA genes were identified by searching vOTUs with tRNAscan-SE v2.0.11 [51], applying both bacterial [parameter: -B] and archaeal [parameter: -A] search modes. Putative host tRNAs that were identical to viral tRNAs were detected among all contigs (excluding self-hits) using blastn v2.13.0 [parameters: -perc_identity 100 -qcov_hsp_perc 100]. Finally, host-associated sequence of prophage contigs, using the CheckV-predicted viral boundaries, were extracted using the *getfasta* command from the BEDTools v2.30.0 software suite [52].

All identified host contigs were taxonomically classified using the Genome Taxonomy Database (GTDB) [53–56] via one of two approaches: if contigs were binned within MAGs, previously assigned GTDB-Tk v2.1.1 [57, 58] classifications for the complete MAGs were used [25]; or otherwise, contigs were classified using CAT v5.2.3 [59] against GTDB r207, which employs a voting algorithm based on taxonomic classifications of every open reading frame (ORF) along a contig. We found that for all contigs that were binned within MAGs, the taxonomic lineage assigned by CAT, at the contig-level, and GTDB-Tk, at the genome-level, were the same, providing validation for the host classifications.

Anti-phage defence systems were identified among the contigs using DefenseFinder v1.0.9 [60, 61], updated with all known anti-phage systems as of January 2023 [defence-finder-models v1.2.2 (https://github.com/mdmparis/defense-finder-models)]. Contigs containing anti-phage systems were taxonomically classified using the same approach as described above.

Alpha-diversity of vOTUs and anti-phage systems associated with each prokaryotic phylum, based on the Shannon index, was calculated using the *diversity* function from the vegan v2.5.7 R package (https://cran.r-project.org/web/packages/vegan/index.html).

### Functional annotation of viral auxiliary metabolic genes

High-quality viral auxiliary metabolic genes (AMGs) were identified using DRAM-v v1.4.3 [62], which stringently considers a gene to be an AMG only if it is flanked by either hallmark viral or viral-like genes. To identify these viral genes, DRAM-v requires VirSorter2 predictions on viral and nonviral genes along contigs. Thus, vOTUs were first re-run with VirSorter2 [parameters: --prep-for-dramv --use-conda-off --viral-gene-enrich-off --keep-original-seq --min-score 0 all]. The vOTU sequences, along with the VirSorter2 viral/nonviral gene predictions, were then used by DRAM-v to identify and functionally annotate AMGs using the default DRAM-v *annotate* and *distill* command parameters. DRAM-v functional annotations of the predicted AMGs are based on an expert-curated AMG database [62], as well as CAZy [63], KOfam [64], Pfam [65], NCBI Viral RefSeq [66], VOGDB (https://vogdb.org/), and MEROPS [67] databases. Genes identified as viral by VOGDB, as CAZymes used for host cell attachment/entry, or as viral-like peptidases, are removed by DRAM-v.

We extended the functional annotations of the identified AMGs by searching against the eggNOG v5 database [68] using eggNOG-mapper v2 [47], and the NCBI nr database (Downloaded: 2023-Jan-18) using blastp v2.13.0 [parameters: -max_hsps 1]. We also screened the AMGs for those involved in phosphorus (P), sulphur (S), and nitrogen (N) cycling against a concatenated database consisting of PCycDB [69], SCycDB [70], and NCycDB [71], using DIAMOND v2.0.15 [72, 73] [parameters: blastp -k 1 -e 1e-5]. For P, S and N cycling genes, we only considered hits with at least 30% amino acid identity over at least 75% of the query sequence. This represents a stricter cut-off than the recommended filtering threshold (30% identity over 25 amino acids [69]), which we set to minimise false-positives.

To identify functionally related metabolic genes across different vOTUs, we clustered AMG protein sequences into orthologous groups using SwiftOrtho [74]. SwiftOrtho first employs an all-to-all homologous search [parameters: -p blastp -e 1e-5 -s 111,111 -a 24], followed by orthology inference [parameters: -c 0.5 -y 0 -a 24], and finally, clustering into orthologous groups of proteins using the graph-based Markov Cluster Algorithm [75] [parameters: -a mcl -I 1.5 -t 24].

### AMG-chemistry correlations and AMG co-occurrences

Pearson’s correlation coefficients (*r*) between the relative abundances of orthologous groups of AMGs (OG-AMGs) and water chemistry concentrations (DOC, Fe, NH_3_, NO_3_^−^, PO_4_^3−^, SO_4_^2−^, salinity and DO) were calculating using the *rcorr* function from the Hmisc v4.6.0 R package (https://cran.r-project.org/package=Hmisc). Only significant correlations (*p* < 0.05, and − 0.8 > Pearson’s *r* > 0.8) were retained for further analysis. It should be noted that viral AMGs might be correlated with water chemistry concentrations due to the presence of other functions encoded by the same vOTU, or due to associations with specific nutrient-cycling bacterial or archaeal hosts. Thus, to reduce the chance of such correlations, we considered only significantly correlated OG-AMGs that were encoded by multiple vOTUs and were associated with multiple host prokaryotic orders. OG-AMGs encoded by prophages were also excluded to ensure correlated metabolic functions were encoded by active viruses.

OG-AMGs that significantly co-occurred on the same vOTU were identified using the cooccur v1.3 R package [76], which calculates co-occurrences that are significantly higher than would be expected by chance using a probabilistic model. Significant pairwise co-occurrences were visualised using the ForceAtlas layout algorithm within Gephi v0.9.7 [77].

## Results and discussion

**Fig. 1:**
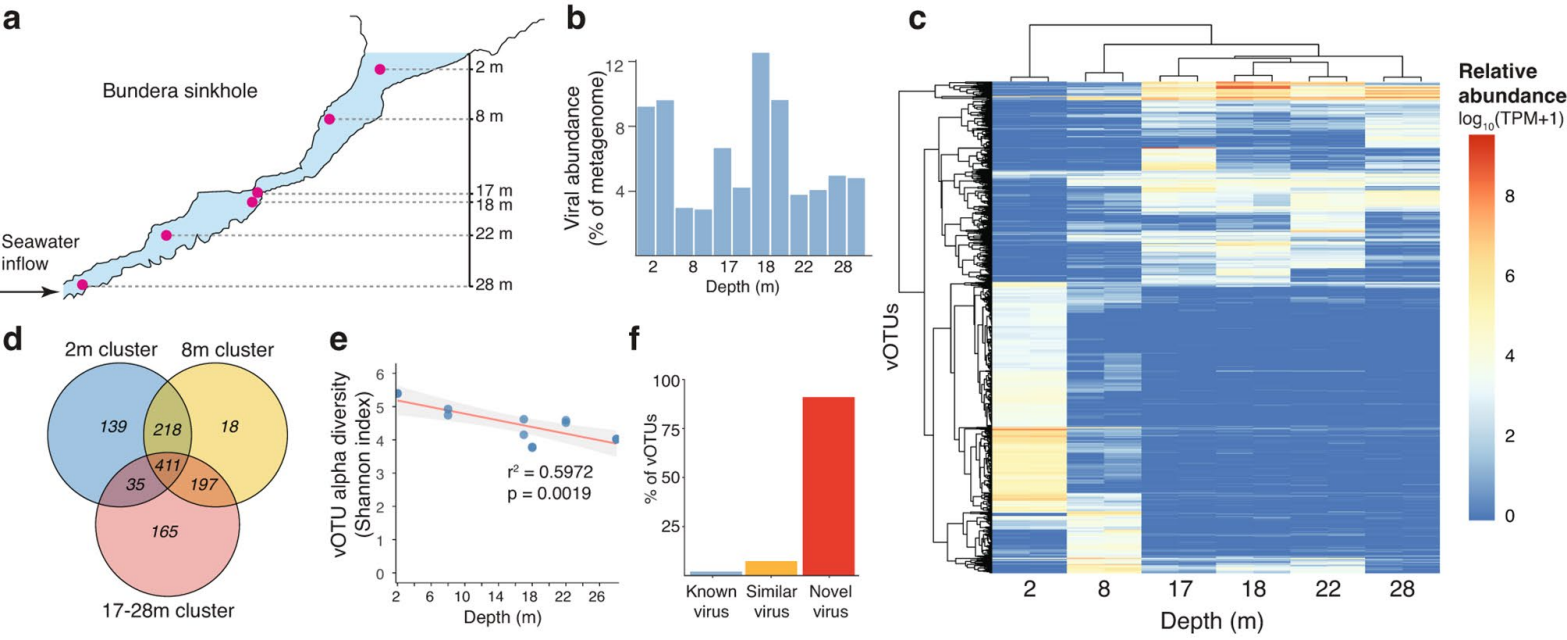
Viral abundance, diversity, and novelty in Bundera Sinkhole. (**a**) Topology of the sinkhole and sampling points for shotgun metagenomic sequencing. Figure panel adapted from Elbourne, et al. [20]. (**b**) Total relative abundance (% of metagenome) of all viruses in each sample, calculated by the transcripts per million (TPM) sum of all viral contigs divided by 1 million (i.e., the TPM sum of all contigs). (**c**) Relative abundance (TPM) of vOTUs within each sample. Colour scale is displayed as log_10_(TPM + 1) to account for TPM values of zero. (**d**) Venn diagram showing the number of unique and shared vOTUs (italicised numbers) among the 2 m (blue), 8 m (yellow), and 17-28 m (red) clusters. (**e**) Relationship between water column depth and vOTU alpha diversity (Shannon index). (**f**) Estimation of viral novelty based on vOTU nucleotide sequence homology with the IMG/VR v3 database [49]. vOTUs were classified based on > 90% identity (known virus), 50–90% identity (similar virus), and < 50% identity (novel virus) over at least 70% of either query or database sequence

We employed shotgun metagenomic sequencing across a water depth profile in Bundera Sinkhole, a dark and low-energy subterranean ecosystem characterised by diverse endemic micro- and macro-organisms, and complex physicochemical profiles. Previous work suggests that a microbial feedback loop, which couples nitrogen and sulphur cycling pathways [25], supports a complex trophic web in the sinkhole. Here, we analysed the viral community in these metagenomic samples to characterise the virus-host-environment interactions in this low-energy chemotrophic environment. We sequenced twelve metagenomic samples from six depths throughout the water column, from 2 to 28 m (Fig. 1a) [25]. In total, we detected 1,183 viral operational taxonomic units (vOTUs), clustered at 95% average nucleotide identity. Of these, the majority (85.45%) were potentially lytic viruses, with only 8.29% classified as proviruses by CheckV [42], and a further 6.26% also considered temperate viruses based on the detection of lysogeny-specific genes (see Methods for genes used). It should be noted, however, that in some cases the lysogeny-specific genes and terminal repeats characteristic of temperate viruses may be on unassembled regions of the viral genomes, and thus may have been misclassified as lytic. Details of vOTUs, including lifestyle predictions, sequence lengths and quality are available as Supplementary Table S4.

### Viruses are highly abundant and novel in Bundera Sinkhole

The relative abundance of viruses in the sinkhole was extremely high, making up as much as 12.5% (average = 6.27%) of a given metagenome after normalising for sequence length of vOTUs and sequencing depth of samples (Fig. 1b). This abundance is particularly high given that samples were collected from 0.22 μm filters prior to DNA extraction, which would likely miss most, if not all, unattached phages (with the caveat being any phages that were stuck to the filter matrix), with the largest known “jumbophage” capsid size being 0.18 μm [33]. This suggests that predominantly phages within or attached to host cells would be captured by the metagenomic sequencing. Viruses attached to particulate matter in the 0.22-60 μm size range might also be included, however, we suspect that there would be a low rate of particle flux in this system owing to minimal surface water input, given the arid climate of the area [26]. Consequently, there is likely less direct input of particles from rainfall runoff or surface water flow. Therefore, since prophages made up only 8% of the vOTUs, the vast majority of sequenced phages were likely involved in active infection during sampling (with the possible exception of pseudolysogens [78] or phage-plasmids [79]). This represents a much greater relative viral abundance than what is usually observed among marine metagenomes, which is generally < 1% of 0.22 μm-filtered seawater samples [80, 81]. Bundera Sinkhole thus represents an environment particularly enriched in actively infecting viruses, suggesting that they play a major role in shaping community dynamics in this ecosystem.

The viral community was stratified along the water column with clear distinctions at different depths (Fig. 1c). We find that vOTU relative abundances form three depth-specific clusters at 2 m, 8 m, and 17-28 m (Fig. 1c). The vOTU composition among the 17–28 m cluster was significantly different to that of the 2 and 8 m clusters (PERMANOVA, *p* = 0.036, adjusted using the Benjamini & Hochberg method [82] for controlling the False Discovery Rate). We observe that both the 2 m and 17-28 m clusters contain a large number of unique vOTUs (Fig. 1d). In contrast, the 8 m cluster is predominantly composed of vOTUs shared with the 2 m and 17-28 m clusters.

This depth-dependent clustering reflects the same stratification of both the physicochemical profile and prokaryote community previously observed in the sinkhole [20, 25], suggesting that the community structure of hosts and viruses, along with the environmental conditions, are tightly linked. Specifically, this stratification reflects phases along salinity and dissolved oxygen gradients (Supplementary Table S1) that appear to be shaping the prokaryotic community [25], which in turn, is likely shaping the viral community. Further, the physicochemical stratification appears to be stable over time, with similar profiles observed between sampling efforts spanning 10 years [20, 28]. This suggests that the microbial stratification patterns might also exhibit similar temporal stability, although longitudinal microbial sampling is needed to confirm this.

High vOTU alpha diversity was observed throughout the water column with Shannon index values ranging from 3.8 to 5.4 (Fig. 1e). Although, we observe a slight but significant decrease (r^2^ = 0.5972, *p* = 0.0019) in vOTU diversity with water depth (Fig. 1e). These highly abundant and diverse vOTUs largely represent undescribed viruses, with only 1.9% matching reference viruses from the IMG/VR v3 database [49] (Fig. 1f). This represents a greater degree of novelty than other reported marine-associated environments, including seawater (comprising ~ 15% known viruses) [83], or marine biofilms (9% known viruses) [84]. Rather, this degree of novelty is consistent with more extreme and less sampled marine environments, such as deep ocean trenches [85, 86], which comprise 1–2% known viruses. Such novelty is also reflected among the prokaryotic community characterised in previous studies of the sinkhole [20, 25], likely driven by adaptation to the distinctive physicochemical properties of anchialine ecosystems.

### Prokaryote host range of viral community

**Fig. 2:**
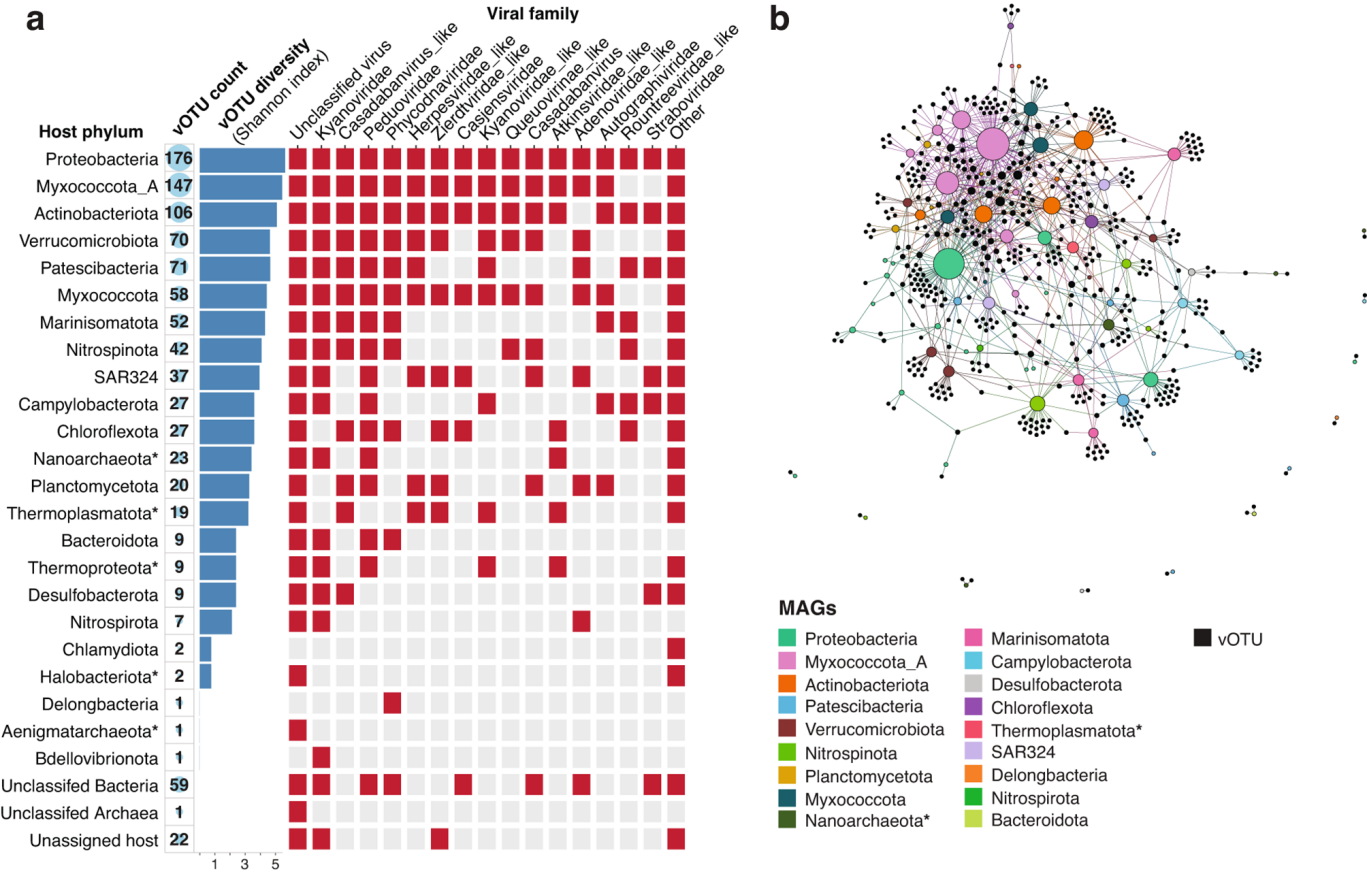
Phage host range predictions using CRISPR spacers, viral tRNA homology and prophage genomic landscape. (**a**) Prokaryotic host phyla of viral community. Archaeal phyla are denoted with asterisks. From left to right: the vOTU count that are predicted to infect each host phylum are displayed by numerals, and represented visually by the size of the circles; vOTU alpha diversity (Shannon index) infecting each host phylum, displayed for all assigned phyla; viral taxonomic classifications (family-level) of the vOTUs infecting each host phylum are displayed by red tiles. (**b**) Network showing links between vOTUs (black nodes) and metagenome-assembled genomes (MAGs; coloured nodes). MAG nodes are coloured by phylum. The size of each MAG node is based on the number of vOTUs it is associated with. The network was visualised using the ForceAtlas layout algorithm within Gephi v0.9.7 [77]

Using an integrated approach of CRISPR spacers, viral tRNA homology and prophage genomic landscape, we could assign a prokaryotic host to 50% of vOTUs at the phylum-level (Supplementary Table S5). This high prediction rate provides an ideal opportunity to assess the ecological and environmental significance of virus-host reactions in this system. The most commonly predicted host phyla were Proteobacteria, Myxococcota_A and Actinobacteriota (Fig. 2a). These phyla were hosts to considerable vOTU diversity, spanning multiple viral families (Fig. 2a). Individual metagenome-assembled genomes (MAGs) from these phyla were often host to multiple vOTUs (Fig. 2b). These bacterial phyla, particularly Proteobacteria and Myxococcota, are also predicted to be largely responsible for mediating nutrient cycles that drive chemotrophic energy production in Bundera Sinkhole [25]. Proteobacteria dominate carbon, nitrogen, and sulphur cycling in this sinkhole, while both Proteobacteria and Myxococcota are functionally coupled, mediating a DNRA (dissimilatory nitrate reduction to ammonia) and nitrification feedback loop in this system [25]. Thus, the large proportion of phages that target ecologically important host taxa highlight their possible role in influencing biogeochemical cycling in this ecosystem, either through modulating host population dynamics and/or their metabolism.

**Fig. 3:**
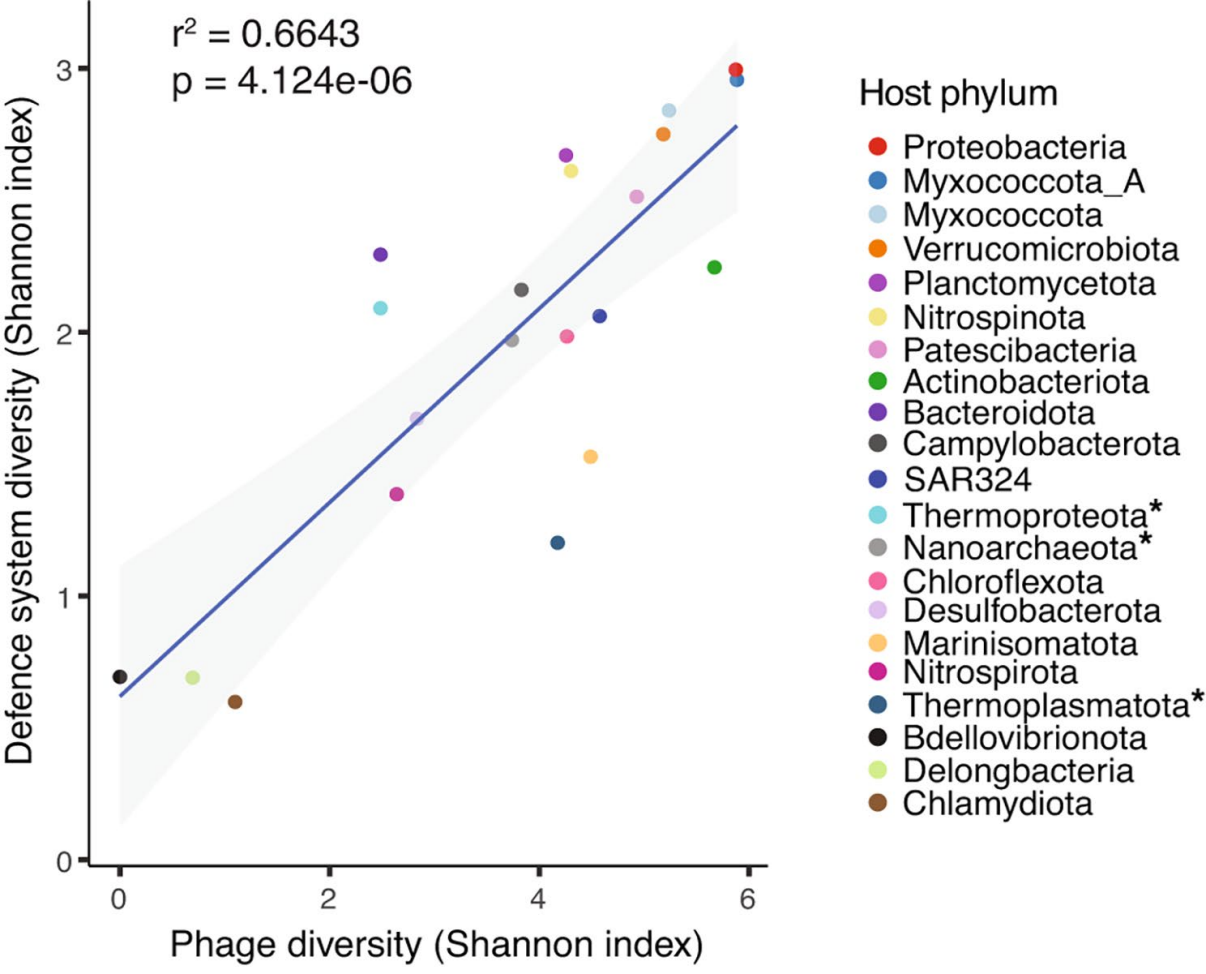
Correlation between within-phylum alpha diversity of phages and defence systems. Alpha diversity of phages and defence systems are estimated using the Shannon index. Points are coloured based on phylum, with archaeal phyla denoted with asterisks. The shaded region represents the 95% confidence interval of the fitted linear model

Likely in response to the high viral abundance and diversity, the prokaryotic hosts collectively encoded a diverse set (*n* = 72) of anti-phage systems, covering more than half of all currently known systems (Supplementary Fig. S1). The diversity of defence systems within each phylum was strongly correlated with the viral diversity that infect that particular phylum (Fig. 3).

### Viral auxiliary metabolic functions may be correlated with biogeochemistry

**Fig. 4:**
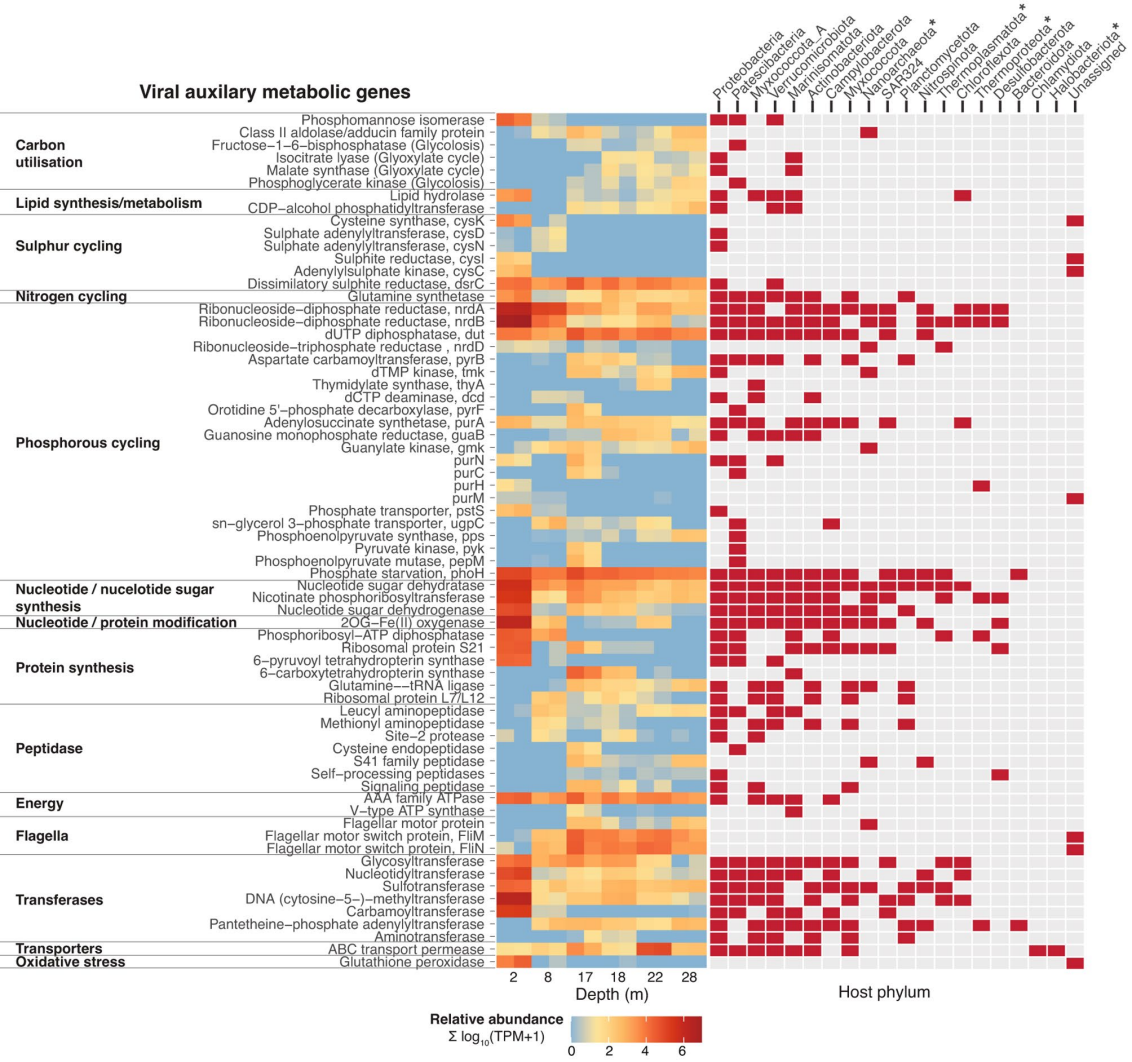
Viral auxiliary metabolic genes. Heatmap (left) displaying the relative abundance (TPM) of detected viral auxiliary metabolic genes (AMGs) in each sample. The host phyla the viruses carrying the detected AMGs are displayed on the right (red tiles). Colour scale is displayed as log_10_(TPM + 1) to account for TPM values of zero. Archaeal phyla are denoted with asterisks

To investigate the potential link of viruses and biogeochemistry, we examined viral auxiliary metabolic genes (AMGs) that were predicted using a highly stringent approach (as outlined in Methods). Together, 701 high-confidence AMGs were detected, encompassing a wide range of metabolic functions (Fig. 4; Supplementary Table S6). Some of the AMGs appear to be depth-specific, e.g., those encoding carbon utilisation enzymes or flagellar motor proteins, which have a greater relative abundance in deeper waters of the sinkhole (Fig. 4). Conversely, subsets of AMGs were highly abundant across all depths, particularly those involved in the biogeochemical cycling of sulphur (e.g., sulphite reduction) and phosphorus (e.g., nucleotide synthesis).

Interestingly, we detected a putative flagellar motor protein predicted to be encoded by a nanoarchaeal virus (Fig. 4). Nanoarchaea are thought to be obligate ectosymbionts relying on host archaea to complete essential metabolic pathways. The flagella of nanoarchaea are thought to facilitate their initial attachment to potential hosts [87]. Thus, the putative viral-encoded flagellar motor protein may assist an infected nanoarchaeal cell in attaching to its own host, which will ultimately promote viral fitness. The possibility of this tripartite interaction warrants further investigation.

AMGs associated with a wider range of host phyla were largely involved in nucleotide/protein synthesis and modification, transferase activities, and transport functions (Fig. 4). As these viral genes are likely expressed by diverse host taxa, such AMG functions are probably important for viral-host dynamics.

**Fig. 5:**
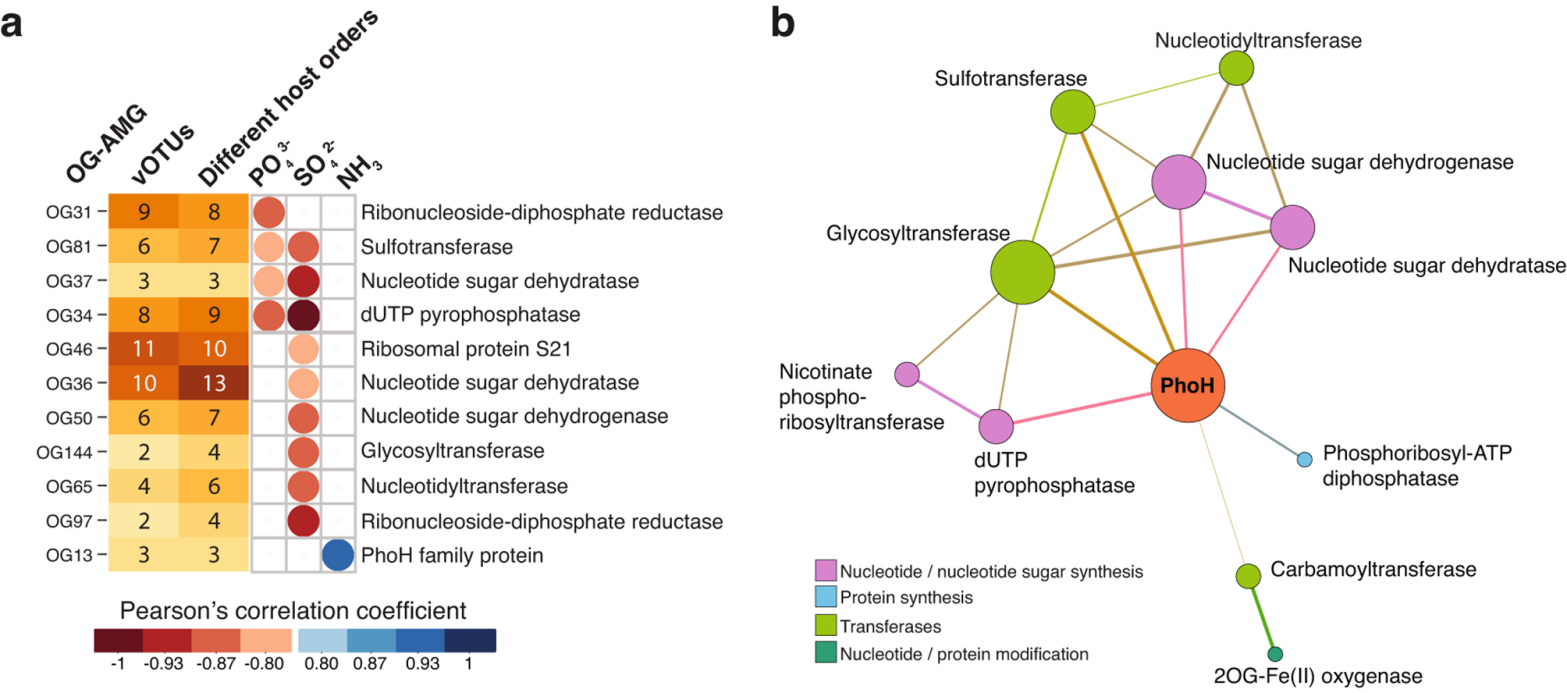
Viral AMG correlations and co-occurrences. (**a**) Orthologous groups of AMGs (OG-AMG) that are significantly correlated (*p* < 0.05, and − 0.8 > Pearson’s *r* > 0.8) with water chemistry profiles. Only OG-AMGs that are encoded by multiple non-prophage vOTUs that are collectively associated with multiple host orders are included. (**b**) Network showing viral AMG functions that significantly (*p* < 0.05) co-occur on the same vOTU more than would be expected by chance. The size of the nodes is governed by the number of co-occurrences, and are coloured based on functional categories. The thickness of connecting lines (edges) represent the degree of co-occurrence significance, with thicker edges representing lower *p*-values (available as Supplementary Table S8)

We found that the relative abundances of several AMGs were associated with the chemical profile of the sinkhole, particularly with phosphate (PO_4_^3−^) and sulphate (SO_4_^2−^) concentrations (Fig. 5a). To minimise correlations caused by high abundance of individual vOTUs, we first clustered protein sequences into functionally orthologous groups of AMGs (OG-AMGs), ensuring that all OG-AMGs spanned across multiple different vOTUs (excluding prophages, which may represent remnants of inactive viruses) and different host orders. We reasoned that if a given functional trait is encoded by distinct and active viruses, that collectively infect multiple prokaryotic taxa, and still exhibited a strong correlation with a given environmental parameter, then it is more likely to represent a genuine association. Using this approach, we found 44 OG-AMGs correlated with both salinity and dissolved oxygen (DO) levels in the surrounding water (Supplementary Table S7). These associations are likely driven by vOTU and host composition, which are shaped by these two environmental parameters.

We found that phosphate and sulphate concentrations displayed strong negative correlations (Pearson’s *r* < − 0.8, *p* < 0.05) with OG-AMGs involved in nucleotide synthesis (ribonucleoside-diphosphate reductases, dUTP pyrophosphatases, nucleotide sugar dehydratases and dehydrogenases), protein synthesis (ribosomal protein S21), and transferase activities (sulfotransferases, nucleotidyltransferases, glycosyltransferases). We propose that these AMGs are largely involved in promoting phage replication by increasing nucleotide and protein production. These processes would raise the host demand for phosphorus (P) and sulphur (S) compounds needed for DNA and protein synthesis and/or modification.

Many of these significantly correlated AMGs, including transferases and those involved in nucleotide/protein synthesis co-occurred together in the same vOTU more than would be expected by chance (*p* < 0.05; Fig. 5b). The observed transferases can have broad functional roles, however, due to their significant co-occurrence with nucleotide/protein synthesis and modification genes, we suspect that they play a role in complex modifications of nucleotides or proteins. Glycosyltransferase- and sulfotransferase-based modification systems involved in nucleotide glycosylation and sulfation have been previously reported in phages [88]. Additionally, many of these genes co-occurred with *phoH* (Fig. 4b). PhoH, named ‘phosphate-starvation inducible protein’, although functionally uncharacterised, has ATP-binding activity and is thought to be a component of the phosphate regulon in *Escherichia coli* [89]. Despite its name, however, *phoH* expression under phosphate limitation appears to vary between bacteria, with phosphate-starvation induction in *E*. *coli* and *Corynebacterium glutamicum*, but not in the marine cyanobacterial genera, *Synechococcus* and *Prochlorococcus* [90–93]. Han, et al. [94] recently proposed that viral-encoded PhoH acts as a nucleotide synthase, binding and hydrolysing ATP to obtain energy for viral nucleotide synthesis, although experimental evidence is yet to validate this hypothesis.

### Virus-host-environment interactions

Although, additional studies, particularly longitudinal studies, are needed to further test the correlations observed in the present study, we hypothesise a model of AMG-mediated virus-host-environment interactions, as described below. Viral AMGs involved in nucleotide synthesis were strongly correlated with phosphate depletion. It has previously been argued that viral AMGs can drive the loss of environmental P [94], however, the direction of this correlation cannot be determined– i.e., viral AMGs are being selected under, or driving, P depletion. The former case would represent AMG-mediated adaptation of viruses to the prevailing environmental conditions, highlighting the dynamic interactions between viruses, their hosts, and the environment. In the latter case, phage-mediated alteration of host metabolism might be altering biogeochemistry and nutrient availability in the surrounding water. This may be particularly pertinent to P cycling, given the huge relative abundance of actively infecting phages in the sinkhole, and their proportionally much greater demand for P (C/N/*P* ≈ 20/6/1) [6] than bacteria (~ 106 − 69/16/1) [95].

Additionally, many of the P metabolism-related nucleotide synthesis AMGs were also strongly correlated with a decrease in sulphate concentrations (Fig. 5a). Here, phage-driven increase in nucleotide synthesis, which would boost viral protein production, would consequently increase host demand for sulphate needed for amino acid synthesis (e.g., cysteine and methionine), or thiol modifications of nucleic acids/proteins [96]. Again, this might represent viral AMGs being selected under, or driving, S depletion. As an adaptive response in the former case, virocells with increased protein production may outcompete uninfected cells for the limited available sulphate. Conversely, increased protein production of the virocells, might itself be driving sulphate depletion in the surrounding environment. If this is the case, it has the potential to significantly impact the ecology of all trophic levels in this system. Microbial chemotrophic utilisation of sulphur has been identified as a key energy source in this low-nutrient environment, which supports diverse and endemic organisms [20, 25–27].

The abundance of viral-encoded ribosomal protein S21 (rpS21) was also significantly correlated with a decrease in environmental sulphate (Fig. 5a), further highlighting the putative link between viral protein production and water sulphate concentrations. In bacteria, rpS21 is essential for translation initiation, with ribosomes incapable of binding mRNA in its absence [97]. This has led to the prediction that the function of phage-encoded rpS21 is to compete with and replace host rpS21 in ribosomes, selecting preferentially for phage mRNA transcripts [98, 99]. Indeed, phage-encoded rpS21 has been found to be co-expressed with core viral structural genes, suggesting it to be useful during late phage infection when large-scale protein production is required for assembly of phage particles [100].

**Fig. 6:**
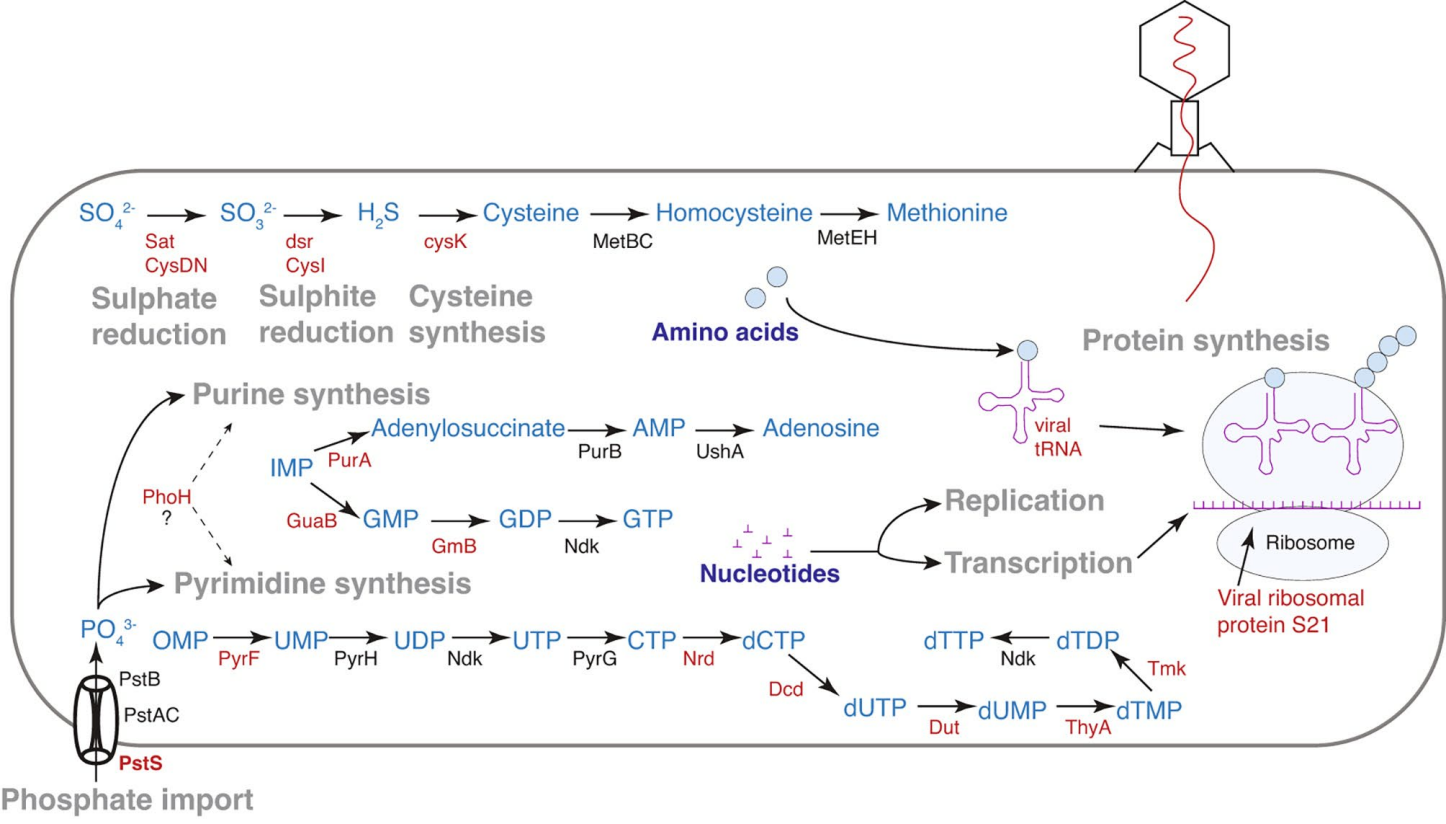
Hypothesised model of virus-host interactions associated with phosphorus and sulphur metabolism. Metabolic reactions are displayed in grey text, with the reaction products and substrates labelled in blue. Viral-encoded proteins and tRNAs are labelled in red text, while host proteins are labelled in black. The model is a conceptual diagram of key reactions in this system as a whole, spanning all sampled depths of the water column

Together, our findings indicate that viral AMG products that can promote protein production are possibly selected under, or driving, the depletion of sulphate and phosphate in this ecosystem. We therefore propose a model of such virus-host-environment interactions, whereby viral AMG products are modulating host metabolism of P and S to promote viral particle production via increased nucleotide and protein synthesis (Fig. 6). Generally, viral AMGs do not encompass all enzymes in a metabolic pathway, but instead encode key rate-limiting steps, or regulators of a pathway [101–103]. We find that, collectively, viruses in this ecosystem encode key enzymes needed for the production of sulphur-containing amino acids, and P metabolic enzymes involved in purine and pyrimidine nucleotide synthesis (Fig. 6).

## Conclusion

Here, we describe and characterise the viral community inhabiting Bundera Sinkhole. We find that viruses are highly abundant and display a high degree of novelty, with less than 2% consisting of described vOTUs. We also predict the prokaryotic hosts of these viruses, finding that the most abundant host phyla represent important taxa likely driving key metabolic processes in this ecosystem. Further, we characterise the metabolic potential encoded by viral AMGs, which are largely involved in promoting nucleotide and protein synthesis and are strongly correlated with declines in environmental phosphate and sulphate. This study represents the first metagenomic investigation of viruses in anchialine ecosystems, and generates novel hypotheses and insights into virus-host-environment interactions in such low-energy environments.

### Electronic supplementary material

Below is the link to the electronic supplementary material.

Supplementary Material 1

Supplementary Material 2

## Data Availability

Raw metagenomic sequence data are available in the NCBI SRA Database under BioSample Accessions SAMN32209613-SAMN32209624, from the BioProject PRJNA911846. MAG and vOTU sequences can be downloaded from figshare (https://figshare.com/projects/Metagenome-assembled_prokaryote_and_viral_genomes_from_Australia_s_only_anchialine_cave_Bundera_Sinkhole/191523). All other data are available as Supplementary Information.
