# Supplementary material for: Exploring virus-host-environment interactions in a chemotrophic-based underground estuary
Source: Environ Microbiome. 2024 Jan 30;19:9. doi: 10.1186/s40793-024-00549-6 (PMC10829341; doi:10.1186/s40793-024-00549-6)
Supplement: Supplementary file 1 — Supplementary Material 1 [file 40793_2024_549_MOESM1_ESM.docx]

**Supplementary Figure**

**Fig. S1.** **Anti-phage defence systems in Bundera Sinkhole.** Diversity of defence systems among prokaryotic phyla. Archaeal phyla are denoted with asterisks. See <https://github.com/mdmparis/defense-finder-models/blob/master/List_system_article.md> for a description of each defence system.
